# Impact of interferon-β and dimethyl fumarate on nonlinear dynamical characteristics of electroencephalogram signatures in patients with multiple sclerosis

**DOI:** 10.3389/fninf.2025.1519391

**Published:** 2025-02-28

**Authors:** Christopher Ivan Hernandez, Natalia Afek, Magda Gawłowska, Paweł Oświęcimka, Magdalena Fafrowicz, Agnieszka Slowik, Marcin Wnuk, Monika Marona, Klaudia Nowak, Kamila Zur-Wyrozumska, Mary Jean Amon, P. A. Hancock, Tadeusz Marek, Waldemar Karwowski

**Affiliations:** ^1^Computational Neuroergonomics Laboratory, Department of Industrial Engineering and Management Systems, University of Central Florida, Orlando, FL, United States; ^2^Doctoral School in the Social Sciences, Jagiellonian University, Kraków, Poland; ^3^Department of Cognitive Neuroscience and Neuroergonomics, Jagiellonian University, Kraków, Poland; ^4^Complex Systems Theory Department, Institute of Nuclear Physics, Polish Academy of Sciences, Kraków, Poland; ^5^Mark Kac Centre for Complex Systems Research, Jagiellonian University, Kraków, Poland; ^6^Department of Neurology, Jagiellonian University Medical College, Kraków, Poland; ^7^Department of Neurology, University Hospital in Krakow, Kraków, Poland; ^8^Centre for Innovative Medical Education, Jagiellonian University Medical College, Kraków, Poland; ^9^Department of Informatics, Luddy School of Informatics, Computing, and Engineering, Indiana University Bloomington, Bloomington, IN, United States; ^10^Department of Psychology, University of Central Florida, Orlando, FL, United States; ^11^Institute for Simulation and Training, University of Central Florida, Orlando, FL, United States; ^12^Faculty of Psychology, SWPS University, Katowice, Poland

**Keywords:** electroencephalogram, complexity, nonlinear dynamics, sample entropy, Higuchi’s fractal dimension, multiple sclerosis

## Abstract

**Introduction:**

Multiple sclerosis (MS) is an intricate neurological condition that affects many individuals worldwide, and there is a considerable amount of research into understanding the pathology and treatment development. Nonlinear analysis has been increasingly utilized in analyzing electroencephalography (EEG) signals from patients with various neurological disorders, including MS, and it has been proven to be an effective tool for comprehending the complex nature exhibited by the brain.

**Methods:**

This study seeks to investigate the impact of Interferon-β (IFN-β) and dimethyl fumarate (DMF) on MS patients using sample entropy (SampEn) and Higuchi’s fractal dimension (HFD) on collected EEG signals. The data were collected at Jagiellonian University in Krakow, Poland. In this study, a total of 175 subjects were included across the groups: IFN-β (*n* = 39), DMF (*n* = 53), and healthy controls (*n* = 83).

**Results:**

The analysis indicated that each treatment group exhibited more complex EEG signals than the control group. SampEn had demonstrated significant sensitivity to the effects of each treatment compared to HFD, while HFD showed more sensitivity to changes over time, particularly in the DMF group.

**Discussion:**

These findings enhance our understanding of the complex nature of MS, support treatment development, and demonstrate the effectiveness of nonlinear analysis methods.

## 1 Introduction

Multiple sclerosis (MS) is a chronic inflammatory disease of the central nervous system (CNS). It is defined by the spread of demyelinating lesions in the CNS over space and time (Siffrin et al., 2010). Neuronal injury occurs early in the disease and is linked to inflammatory activity. The remaining stages of neuronal damage after focal axonal lesions include axon degeneration and atrophy of neuronal cell bodies and dendrites (Siffrin et al., 2010). Atrophy and long-term disability in patients with MS can be attributed to the loss of neurons and their processes. Since inflammation is one of the leading causes of neurodegeneration, a combination of neuroprotective agents and anti-inflammatory treatments are encouraged early on Siffrin et al. (2010).

There are several treatments for multiple sclerosis; however, this paper will focus on two treatments widely used in managing this disease: Interferon-β (IFN-β) and dimethyl fumarate (DMF) (Reick et al., 2014). There are three main types of Interferon: Interferon-alpha, Interferon-beta, and Interferon-gamma (Jakimovski et al., 2018). Interferon-β treats different types of MS by reducing inflammation and regulating the immune response. This drug is administered via injection, and common side effects include flu-like symptoms, injection-site reactions, myalgia, depression, and increased liver enzymes (Jakimovski et al., 2018). Dimethyl fumarate is branded as Tecfidera®. Also known as B-12, it is an oral medication that regulates the immune system and prevents stress and inflammation by activating the nuclear factor erythroid 2-related pathway. Some side effects include gastrointestinal issues, flushing, and lymphopenia (Linker and Haghikia, 2016; Mills et al., 2018).

It is important to note that Sattarnezhad et al. (2022) recognized that patients on IFN-β experienced a higher occurrence of relapses and a higher number of magnetic resonance imaging (MRI) lesions. In contrast, those on dimethyl fumarate experienced a lower occurrence of relapses and a lower number of lesions (Sattarnezhad et al., 2022). D’Amico et al. (2021) also observed fewer relapses in dimethyl fumarate compared to IFN-β (D’Amico et al., 2021). To further back this up, Lorscheider et al. (2021) demonstrated that dimethyl fumarate had similar efficacy compared to another drug, fingolimod, and Cohen et al. (2010) proved fingolimod had a better performance than IFN-β (Lorscheider et al., 2021; Cohen et al., 2010). Table 1 shows a summary of the characteristics of IFN-β and DMF outlined in several studies (Cohen et al., 2010; D’Amico et al., 2021; Linker and Haghikia, 2016; Lorscheider et al., 2021; Mills et al., 2018; Jakimovski et al., 2018; Sattarnezhad et al., 2022).

**Table 1:** Summary of interferon-β vs. dimethyl fumarate.

Interferon-β (IFN-β)	Dimethyl fumarate (DMF)
Injection	Oral
Helps reduce inflammation and regulates the immune response	Regulates the immune system and prevents stress and inflammation
Side effects: flu-like symptoms, injection site reactions, myalgia, depression, and an increase in liver enzymes	Side effects: gastrointestinal issues, flushing, and lymphopenia
Higher occurrence of relapses	Lower occurrence of relapses
Higher number of MRI lesions	Lower number of MRI lesions

Many illnesses exhibit irregular brain wave activity, including MS, which can be detected and analyzed by electroencephalography (EEG) (Sanei and Chambers, 2007). Structural changes observed in the brain wave activity of MS patients can be identified by EEG analysis, as opposed to imaging methods, such as MRI (Carrubba et al., 2012). Despite appearing random, EEG signals exhibit complex characteristics with intricate temporal organization and are fundamentally deterministic (Rodriguez-Bermudez and Garcia-Laencina, 2015; Pritchard and Duke, 1995). Nonlinear analysis methods have successfully captured the complexities and nonlinearities in EEG signals, as opposed to conventional linear methods, such as autocorrelation (Rodriguez-Bermudez and Garcia-Laencina, 2015; Pritchard and Duke, 1995; Kargarnovin et al., 2023). Sample entropy (SampEn) and fractal dimension analysis are both commonly used to analyze the complexity or irregularity of a signal, particularly in nonlinear contexts, and we opted to use both sample entropy and Higuchi’s fractal dimension (HFD) in our study (Kargarnovin et al., 2023; Hernandez et al., 2023).

Among the algorithms used for entropy estimation, particularly concerning EEG data, SampEn has been successfully employed (Bruce et al., 2009; Cuesta-Frau et al., 2017; Zhang et al., 2021). Created to reduce the bias of approximate entropy (ApEn), SampEn quantifies time series data regardless of the signal length, providing insights into complexity, irregularity, and rate at which new information is produced, making it especially valuable in analyzing noisy signals (Duran et al., 2013; Richman and Moorman, 2000). Studies have analyzed EEG signatures using SampEn, and a couple to note are studies conducted by Mohseni and Moghaddasi (2022) and Shalbaf et al. (2012). In Mohseni and Moghaddasi (2022), SampEn was used to develop a diagnostic tool for MS, and their tool attained significantly higher diagnostic activity compared to other MS diagnostic methods (Mohseni and Moghaddasi, 2022). Shalbaf et al. (2012) used SampEn to measure the effects of sevoflurane on electroencephalogram, and they concluded it outperformed response entropy (RE) (Shalbaf et al., 2012).

Fractal dimension (FD) is a common measure of time series regularity, widely used to quantify long-range correlation and power law dependencies by determining the scaling exponent. FD has demonstrated its ability to differentiate between healthy and pathological brains, indicating its strength in examining the maturation and degeneration of brain function (Marino et al., 2019; Smits et al., 2016; Zappasodi et al., 2014; Zappasodi et al., 2015). Marino et al. (2019) noted that changes in FD can reflect an alteration in the complexity of the dynamical nature of the brain, and it could be potentially tied to cognitive or perceptual impairment, as seen in studies investigating dementia and Alzheimer’s symptoms (Zappasodi et al., 2015; Marino et al., 2019; Ahmadlou et al., 2011; Smits et al., 2016). Higuchi’s fractal dimension (HFD) is the most accurate in estimating FD compared to other FD methods (Esteller et al., 2001; Raghavendra et al., 2009; Kesić and Spasić, 2016). It has been a prominent method in analyzing neuronal data, such as EEG and electrocorticography (ECoG), because it holds advantages over linear and spectral analysis methods due to its speed, accuracy, and computational cost (Paramanathan and Uthayakumar, 2008; Spasic et al., 2011; Chouvarda et al., 2011; Arle and Simon, 1990). In some cases, HFD produces better results when combined with other linear and nonlinear methods (Kesić and Spasić, 2016).

Thus, a research gap lies in investigating the nonlinear dynamics in EEG signals from multiple sclerosis patients under different drug treatments, such as IFN-β and DMF. This study aims to compare the nonlinear dynamics of EEG signals between MS patients treated with IFN-β and DMF. The following research questions were developed prior to the study:

- RQ1: Does the EEG of patients with MS exhibit increased complexity compared to the control group?
- RQ2: How do the complexity characteristics of EEG signals differ between MS patients undergoing treatment with IFN-β and those treated with DMF?
- RQ3: Which complexity measure is most sensitive to the effects of IFN-β or DMF treatment on EEG dynamics in MS patients?
- RQ4: Can the observed changes in complexity characteristics of EEG signals be used as potential biomarkers for monitoring the effectiveness of IFN-β or DMF treatment in MS patients?

In response to each research question, we hypothesize the following:

1. EEG data collected from patients with MS demonstrates an increase in complexity when compared to healthy participants, as reflected via sample entropy and Higuchi’s fractal dimension.
2. Sample entropy and Higuchi’s fractal dimension, will illustrate distinguishable alterations between patients treated with IFN-β, patients treated with DMF, and the control group (healthy participants). Patients treated with DMF will exhibit significant differences in nonlinear characteristics compared to patients treated with IFN-β and the control group.
3. Sample entropy will demonstrate the highest sensitivity and the greatest predicted value in evaluating the effects of IFN-β or DMF treatment on MS compared to the control group.
4. Nonlinear analysis of EEG signals via sample entropy and Higuchi’s fractal dimension will reveal significant and consistent changes over time in MS patients undergoing IFN-β and DMF treatments relative to the control group of healthy patients. This will serve as definitive biomarkers for assessing treatment effectiveness and disease progression.

## 2 Methodology

### 2.1 Location of data collection and participants

The data were collected at Jagiellonian University in Krakow, Poland. The study included two groups of subjects: patients with early onset relapsing–remitting multiple sclerosis (RRMS) and healthy subjects. In the group of MS patients, there were two subgroups: those treated with IFN-β and those treated with DMF. The total number of participants for this analysis is 175. To further break it down, 39 patients were on IFN-β, 53 were on DMF, and there were 83 healthy controls. The IFN-β group consisted of participants between 22 and 63 years old (*M* = 39.15, SD = 7.909), and there were 24 females (61.5%) and 15 males (38.5%). The DMF group contained participants between 18 and 54 years old (*M* = 32.11, SD = 7.250). This group had 33 females (62.3%) and 20 males (37.7%). The participants in the control group were between 21 and 61 years old (*M* = 36.22, SD = 8.498). There were 53 females (63.9%) and 30 males (36.1%). There were two rounds of data collection (first measurement and second measurement). The data for the second measurement were obtained 1 year after the data for the first measurement were collected. MS patients’ Expanded Disability Status Scale (EDSS) scores (Kurtzke, 1983) ranged from 1 to 4 in the first measurement and from 1 to 4.5 in the second measurement. The number of relapses in the year prior to each measurement ranged from 0 to 2. A Wilcoxon signed-rank test indicated that there was no significant difference between EDSS scores in the first and second measurements, *z* = −0.958, *p* = 0.338. The median EDSS score was 1 in both the first and second measurements. Similarly, there was no significant difference in the number of relapses in the year prior to each measurement between the first and second measurements, *z* = −0.915, *p* = 0.360. The median number of relapses in the year prior was 0 in both the first and second measurements. The control group did not undergo a second round of data collection because there should not be significant changes in resting state EEG in healthy subjects within 1 year (Kondacs and Szabó, 1999).

### 2.2 Experimental protocol

For this study, data were collected during a resting state task. The resting state task included a six-minute procedure without any stimuli. In the first 3 minutes, subjects were asked to have their eyes open while focusing on a fixation point, and they had to keep their eyes closed in the last 3 minutes. Commands were pre-recorded and played by speakers. A 256-channel dense array EEG system (HydroCel Geodesic Sensor Net, EGI System 300; Electrical Geodesic Inc., OR, USA) was used to collect the data. The researchers decided to remove channels located on the cheeks (E225, E226, E227, E228, E229, E230, E231, E232, E233, E234, E235, E236, E237, E238, E239, E240, E241, E242, E243, E244, E245, E246, E247, E248, E249, E250, E251, E252, E253, E254, E255, and E256) due to many artifacts of low interest in the signal.

### 2.3 Pre-processing

The EEG data underwent pre-processing using MATLAB’s EEGLAB software to ensure data quality and integrity (Delorme and Makeig, 2004). The initial pre-processing stage involved discarding 5 seconds of data that followed sound commands—eliminating these potential artifacts or confounding effects because the experimental instruction allowed for a more precise analysis of the EEG signals. A high pass filter was employed to exclude any signals below the frequency of 0.5 Hz. Adding on, a notch filter to remove power line interference and its harmonics was integrated to reject 50 Hz and its multiplicities from the signal. Independent component analysis (ICA) was conducted. Fifty principal components were used for the analysis to identify and reject artifact components, such as components related to eye movements, muscle activity, or other sources of artifact. Every removed channel was interpolated to estimate the missing values based on surrounding electrodes and provide comprehensive coverage of all channels. Each subject had a sampling rate of 250 Hz for this study.

### 2.4 Autocorrelation

A commonly used linear analysis with applications in neurophysiological data, lag-1 autocorrelation (AC1), was carried out to validate the use of nonlinear analysis (Meisel et al., 2017; Scheffer et al., 2009). AC1 is a reliable measure of the rate at which the autocorrelation function decays (Huang et al., 2018). The autocorrelation function (ACF) is defined in Equation 1, where $x\left(t\right)$ represents the envelope signals, *N* is the length, μ is the mean, and *v* is the variance:


(1) $$ACF\left(s\right)=\overset{N-s}{\underset{t=1}{\sum }}\frac{\left(x\left(t\right)-\mu )(x\left(t+s\right)-\mu \right)}{v},s=1,\ldots ,\frac{N}{2}$$

To obtain lag-1 autocorrelation, we set *s* = 1 (Meisel et al., 2017). Higher AC1 values indicate greater predictability in the signal, whereas lower AC1 values suggest less predictability (Huang et al., 2018).

### 2.5 Sample entropy

Sample entropy (SampEn), initially developed by Richman and Moorman (2000) to measure regularity, was used to analyze the EEG signals across all groups (Duran et al., 2013; Richman and Moorman, 2000). Greater entropy values indicate that the system is complex, irregular, and unpredictable, often associated with a healthy system. Conversely, low entropy values indicate a more deterministic and predictable system, meaning the EEG signals show more regular patterns and less complexity (Duran et al., 2013; Pincus, 2006; Delgado-Bonal and Marshak, 2019). Two notable parameters are used in calculating SampEn: *m* and *r*. The parameter *m* represents the length of the subseries, and *r* represents the similarity criterion (Ramdani et al., 2009). Following the guidance of Costa et al. (2005) and Duran et al. (2013) selected *m* = 2 and *r* = 0.15 as the parameters, and it was noted that the selection of the parameters does not negatively impact the overall pattern of the results (Costa et al., 2005; Duran et al., 2013). Thus, others typically default to the parameters Duran et al. (2013) used, as they are considered standard and, therefore, were deemed appropriate for this study. Following the guidance outlined by Ramdani et al. (2009), the equation for sample entropy is as follows (Richman and Moorman, 2000; Ramdani et al., 2009):

With time series *x*_1_, *x*_2_, … *x*_N,_ subsequences of length *m* are first defined in Equation 2:


(2) $$y_{i}\left(m\right)=\left(x_{i},x_{i+1},\ldots ,x_{i+m-1}\right),\mathrm{where}\;i=1,2,\ldots ,N-m+1$$

After, the quantity is calculated by the following:


(3) $$B_{i}^{m}\left(r\right)=\frac{1}{N-m-1}\overset{N-m}{\underset{j=1,j\neq i\;}{\sum }}\Theta \left(r-||y_{j}\left(m\right)-y_{i}\left(m\right)||\infty \right)$$

The Heaviside function is defined by Θ , and ||·||∞ represents the maximum norm, which is $y_{j}\left(m\right)-y_{i}\left(m\right)\infty =\max_{0\leq k\leq m-1}|X_{j+k}-X_{i+k}|$ . To explain, Equation 3 calculates the sum of the quantity of vectors, $y_{j}\left(m\right)$ , that are within the radius, *r*, from $y_{i}\left(m\right)$ that exist in the reconstructed phase space. Identical matches are excluded and are represented by j≠i . Also, *N – m* represents the total amount of vectors in the (*m* + 1) dimensional state space.

Equation 4 calculates the density:


(4) $$B^{m}\left(r\right)=\frac{1}{N-m}\overset{N-m}{\underset{N-m}{\sum }}B_{i}^{m}\left(r\right)$$

Calculations in the (*m* + 1) space to extend the template matching process are as follows:


(5) $$A_{i}^{m}\left(r\right)=\frac{1}{N-m-1}\overset{N-m}{\underset{j=1,j\neq i\;}{\sum }}\Theta \left(r-||y_{j}\left(m+1\right)-y_{i}\left(m+1\right)||\infty \right)$$

(6) $$A^{m}\left(r\right)=\frac{1}{N-m}\overset{N-m}{\underset{N-m}{\sum }}A_{i}^{m}\left(r\right)$$

In Equation 5, the number of sequences $y_{j}\left(m+1\right)$ within radius *r* of $y_{i}\left(m+1\right)$ is calculated, with the term $y_{j}\left(m+1\right)-y_{i}\left(m+1\right)$ representing the maximum difference between the two subsequences. After calculating the individual template matches $A_{i}^{m}\left(r\right)$ , they are all averaged across all vectors to give $A^{m}\left(r\right)$ , as shown in Equation 6. Then, the total amount of template matches in a *m*-dimensional and *m* + 1 dimensional phase space with *r* is represented by Equations 7 and 8:


(7) $$B\left(r\right)=\frac{1}{2}\left(N-m-1\right)\left(N-m\right)B^{m}\left(r\right)$$

(8) $$A\left(r\right)=\frac{1}{2}\left(N-m-1\right)\left(N-m\right)A^{m}\left(r\right)$$

The sample entropy can then be calculated as follows in Equation 9:


(9) $$\mathrm{SampEn}\left(m,r,N\right)=-\log\left(\frac{A\left(r\right)}{B\left(r\right)}\right)$$

The sample entropy MATLAB script provided by Richman and Moorman (2000) was used in conjunction with an unpublished modified script from Amon (2021) to conduct the analysis (Richman and Moorman, 2000; Amon, 2021).

### 2.6 Higuchi’s fractal dimension

Higuchi’s fractal dimension (HFD) was also employed to analyze the EEG signals. It is another method frequently used in nonlinear analysis, and it details the time series’ complexity and self-similarity (Accardo et al., 1997). Following the outline of the computation summarized in Hernandez et al. (2023), the calculation of HFD involves analyzing a time series data sequence, denoted as *X* (1), *X* (2), …, *X* (*N*), where *N* represents the total number of samples (Hernandez et al., 2023). The selection of a scale factor, *m*, begins the process. This scale factor, *m*, defines the length of the subseries under investigation. The selection of *k* is also necessary to commence the process, as this is the index of the subseries. The cumulative length, L(*m, k*), is calculated by comparing the absolute differences between adjacent data points within the subseries, as shown in Equation 10 (Porcaro et al., 2020):


(10) $$L_{m}\left(k\right)=\frac{1}{k}\left[\underset{i=1,int\left(\frac{N-m}{k}\right)}{\sum }|\begin{array}{l} X\left(m+ik\right)\\ -X\left(m+\left(i-1\right)k\right) \end{array}|.\frac{N-1}{int\left(\frac{N-m}{k}\right)}\right]$$

N is the length of the original time series *X* and $\frac{N-1}{int\left(\frac{N-m}{k}\right)}$ normalizes the function. The average cumulative length across all subseries is calculated to acquire $L\left(k\right)$ , the average length for the given scale factor, as represented in Equation 11:


(11) $$L\left(k\right)=\frac{\sum_{m=1}^{k}L_{m}\left(k\right)}{k}$$

The Higuchi’s fractal dimension is then computed by taking the logarithm of $L\left(k\right)$ , as defined in Equation 12:


(12) $$FD=\frac{\ln\left(L\left(k\right)\right)}{\ln\left(1/k\right)}\;for\;k=1,2,\ldots ,k_{\text{max}}$$

The resulting fractal dimension value represents the fractal dimension of the time series, providing insight into its complexity (Porcaro et al., 2020). The method for calculating Higuchi’s fractal dimension was adopted from Jesús Monge-Álvarez^1^.

Typically, the fractal dimension ranges between 1 and 2, where higher HFD values indicate greater complexity and lower values suggest reduced complexity (Accardo et al., 1997; Scarpa et al., 2017).

**Figure 1:**
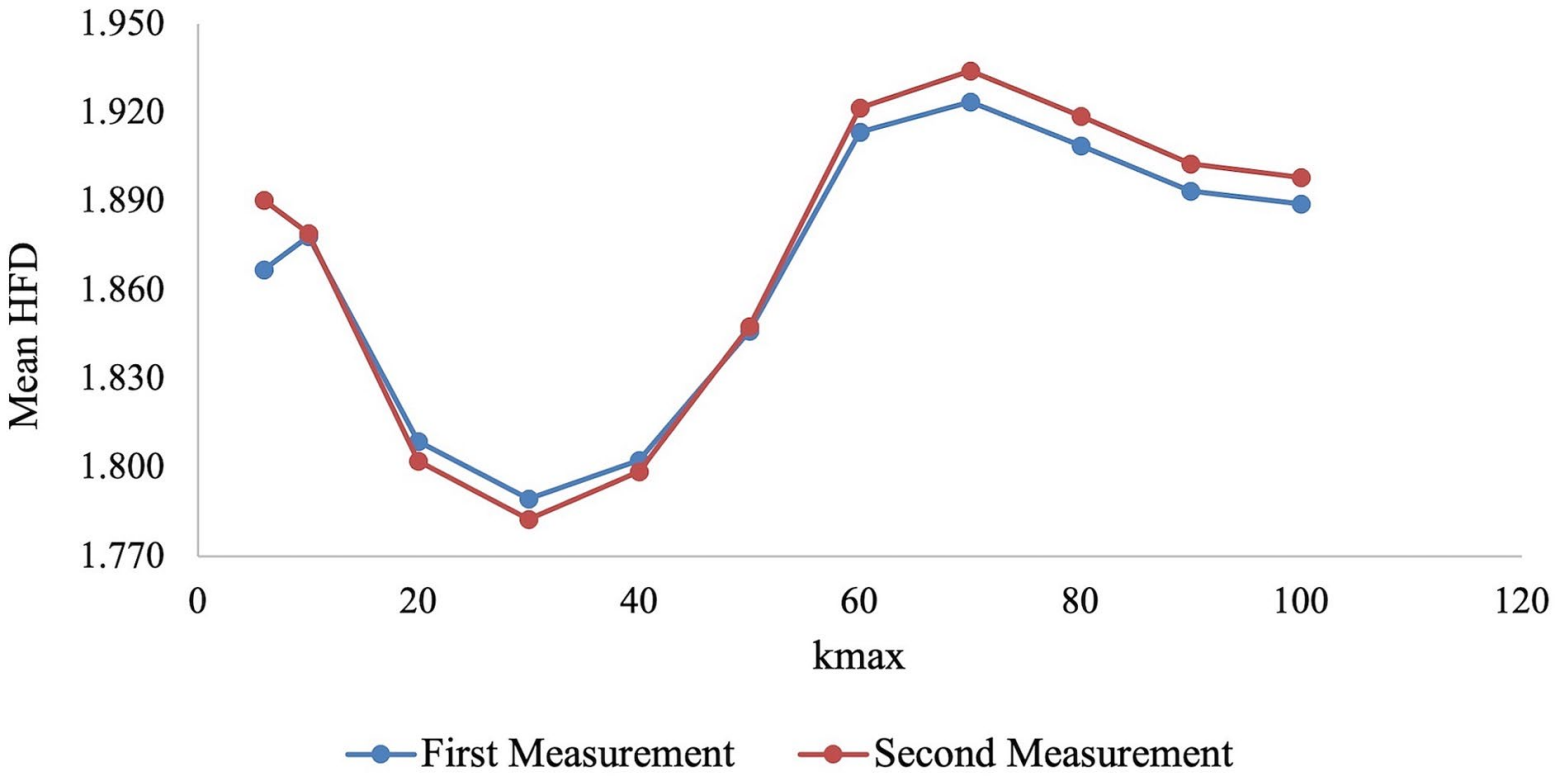
The mean Higuchi’s fractal dimension of the first and second measurements is plotted for each k_max_ to assess where it approaches a local maximum or asymptote.

Currently, no standard method is used to select the most appropriate value for the k_max_ parameter (Kesić and Spasić, 2016). The method selected in this paper is a common method used by Doyle et al. (2004) and Wajnsztejn et al. (2016). They considered the most appropriate k_max_ parameter to be where HFD approaches a local maximum or asymptote (saturation point) (Wanliss et al., 2021; Doyle et al., 2004; Wajnsztejn et al., 2016). According to Figure 1, the data reaches a local maximum at k_max_ = 70. Therefore, k_max_ = 70 was the parameter chosen for this study.

### 2.7 Windowing

For the analysis, each participant’s EEG signal was divided into short 15-s time windows with 50% overlap. This was decided after following the advice of several articles that have opted to divide EEG signals into short time windows for computational efficiency (Mohseni and Moghaddasi, 2022; Ramanand et al., 2004; Er et al., 2021; Kesić and Spasić, 2016). The 50% overlap was chosen to prevent any discontinuity at the frame’s beginning or end (Er et al., 2021).

### 2.8 Statistical analysis

Several statistical analysis techniques were used to understand the data and answer the research questions comprehensively. Descriptive statistics provided a summary of the data. Levene’s and Mauchly’s tests were conducted to test for homogeneity and sphericity. Although homogeneity was violated in most cases, it was not violated in the second measurement of AC1. There was no indication of a violation of sphericity. Given the sample size (*n* > 30) and following guidance from Hair et al. (2010) and Byrne (2010), parametric tests were utilized, as skewness (between −2 and + 2) and kurtosis (between −7 and + 7) were within acceptable ranges (Hair et al., 2010; Byrne, 2010). A paired samples t-test was used to compare the means within subjects, and mixed analysis of variance (ANOVA) was used to investigate the main effects of time and group. Welch’s ANOVA was employed to analyze the means between subjects to address the violation of homogeneity, and standard ANOVA was used to evaluate the means between subjects in the second measurement of AC1, where homogeneity was not violated. Games-Howell *post hoc* test was completed to identify which groups demonstrated significant differences. An alpha level of 0.05 was used as the threshold for determining the effect’s significance.

## 3 Results

### 3.1 Assessment of linearity

Lag-1 autocorrelation (AC1) was carried out to assess the linearity of the dataset. The mean AC1 value of the IFN-β group was 0.800 (SD = 0.044) in the first measurement and 0.815 (SD = 0.042) in the second measurement. For the DMF group, the mean AC1 value was 0.812 (SD = 0.052) in the first measurement and 0.805 (SD = 0.050) in the second measurement. The mean AC1 of the control group was 0.806 (SD = 0.034). Paired samples t-test revealed no significant differences in the means within the IFN-β group (*t*(38) = −1.676, *p* = 0.102) and DMF group (*t*(52) = 0.901, *p* = 0.372). According to the mixed factorial ANOVA, time did not have a significant effect, *F*(1, 172) = 0.727, *p* = 0.395. However, a significant interaction effect of time and group was reported *F*(2, 172) = 3.396, *p* = 0.036, highlighting a significant change in the pattern over time across groups. Due to the violation of homogeneity in the first measurement, *F*(2, 172) = 3.344, *p* = 0.038, Welch’s ANOVA was conducted for between-subjects comparison. No significant differences were reported in the first measurement, *F*(2, 82.498) = 0.651, *p* = 0.524. Since the data in the second measurement, F(2, 172) = 1.636, *p* = 0.198, did not violate homogeneity, standard ANOVA was carried out. Like in the first measurement, no significant differences were reported, *F*(2, 172) = 0.728, *p* = 0.484.

### 3.2 Assessment of nonlinearity

**Figure 2:**
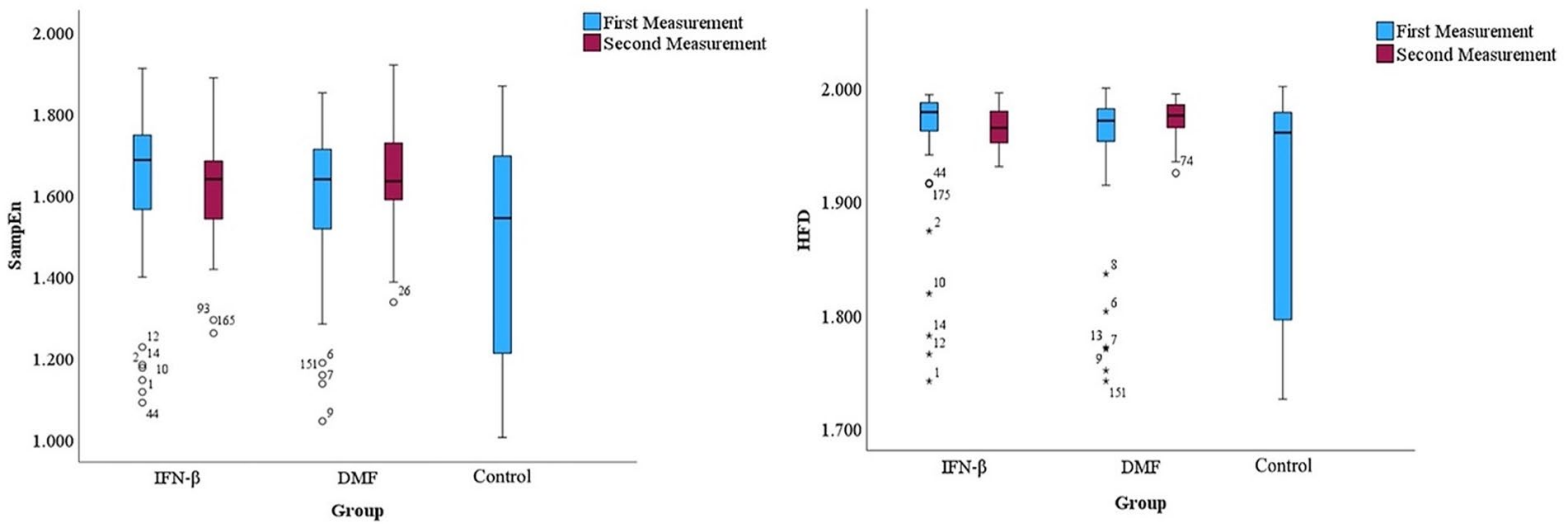
Box plots represent the distribution of SampEn and HFD values across groups.

**Figure 3:**
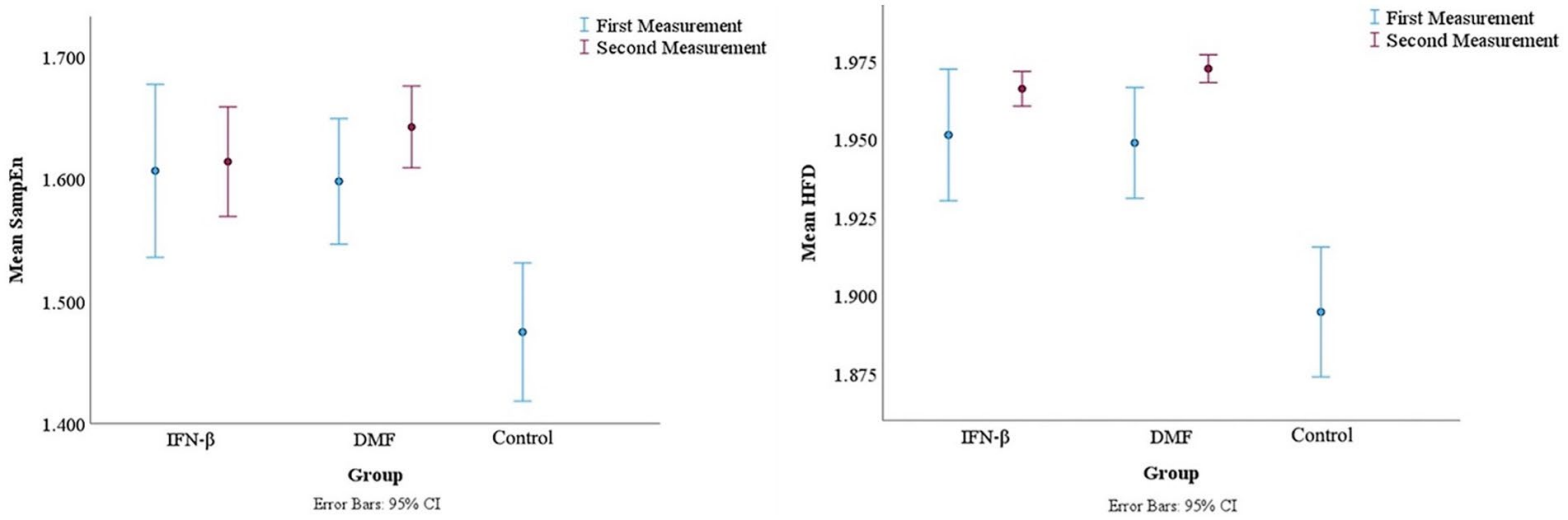
Mean SampEn and HFD for each group with associated error bars.

To assess the complexity of the EEG data, box plots with 95% confidence intervals were created to understand the distribution and central tendency of the SampEn and HFD values across different groups and measurements (Figure 2). Referring to the point plots in Figure 3, both treatment groups at each measurement had recorded relatively high mean SampEn values and HFD values compared to the control group. Summary statistics are shown in Table 2. A paired samples *t*-test was employed to evaluate the significance of the difference within each treatment group.

**Table 2:** Descriptive statistics for SampEn and HFD across groups.

Measurement	Group	N	Mean	SD	Median	IQR	Min	Max
SampEn first measurement	Control	83	1.475	0.259	1.544	0.499	1.005	1.869
	IFN-β	39	1.607	0.219	1.687	0.193	1.090	1.912
	DMF	53	1.598	0.187	1.640	0.206	1.045	1.852
SampEn second measurement	Control	-	-	-	-	-	-	-
	IFN-β	39	1.614	0.138	1.640	0.169	1.261	1.889
	DMF	53	1.643	0.121	1.635	0.154	1.337	1.920
HFD first measurement	Control	83	1.895	0.095	1.960	0.185	1.726	2.001
	IFN-β	39	1.951	0.065	1.979	0.027	1.741	1.994
	DMF	53	1.949	0.064	1.971	0.030	1.742	2.000
HFD second measurement	Control	-	-	-	-	-	-	-
	IFN-β	39	1.966	0.017	1.965	0.028	1.931	1.996
	DMF	53	1.973	0.016	1.976	0.021	1.925	1.995

#### 3.2.1 Variations and trends in sample entropy across groups

The median, interquartile range (IQR), and potential outliers of SampEn are shown in Figure 2 for both time measurements across groups. For the IFN-β group, the median SampEn at the initial measurement was reported as 1.687 (IQR 1.561–1.754), and the median SampEn at the second measurement slightly decreased to 1.640 (IQR 1.516–1.685). Similarly, for the DMF group, the median SampEn at the first measurement was 1.640 (IQR 1.515–1.721), and a slight decrease in median SampEn was observed in the second measurement at 1.635 (IQR 1.578–1.731). The median SampEn for the control group for the first measurement was 1.544 (IQR 1.201–1.699). The presence of outliers confirms the violation of homogeneity.

Referring to Figure 3, only a slight increase in mean SampEn was observed from the first measurement to the second measurement in the IFN-β and DMF groups. Results indicate that the increase in the mean SampEn of the IFN-β treatment group observed in the second measurement (*M* = 1.614, SD = 0.138) was not significant compared to the mean SampEn of its initial measurement (*M* = 1.607, SD = 0.219), *t*(38) = −0.186, *p* = 0.854. For DMF, the mean SampEn of its second measurement (M = 1.643, SD = 0.121) did not differ significantly from its initial measurement (*M* = 1.598, SD = 0.187), *t*(52) = −1.687, *p* = 0.098. The mean SampEn value for the control group was 1.475 (SD = 0.259).

#### 3.2.2 Variations and trends in Higuchi’s fractal dimension across groups

Figure 2 shows the median, interquartile range (IQR), and potential outliers for both measurements across groups for HFD. The median HFD value in the first measurement of the IFN-β group was high at 1.979 (IQR 1.961–1.988), and it saw a minor decrease in the second measurement with a value of 1.965 (IQR 1.951–1.980). In the DMF group, the median HFD value was also high at 1.971 (IQR 1.952–1.982), and an increase in HFD was reported in the second measurement with a value of 1.976 (IQR 1.965–1.986). For the control group, the median HFD value was 1.960 (IQR 1.794–1.979). Like in SampEn, the presence of outliers confirms the violation of homogeneity.

Small increases in mean HFD measurements were observed between measurements in both treatment groups (Figure 3). The mean HFD value in the second measurement of the IFN-β group (*M* = 1.966, SD = 0.017) slightly increased when compared to the first measurement (*M* = 1.951, SD = 0.065); however, it was not significant, *t*(38) = −1.372, *p* = 0.178. On the other hand, the second measurement of the DMF group (*M* = 1.973, SD = 0.016) significantly increased when compared to the first measurement (*M* = 1.949, SD = 0.064), *t*(52) = −2.760, *p* = 0.008. The significant results are shown in Table 3. The mean HFD value for the control group was 1.895 (SD = 0.095).

**Table 3:** Paired samples *T*-test for HFD in the DMF treatment group.

Group	*t*	df1	Two-sided *p*
DMF	−2.760	52	0.008

### 3.3 Longitudinal analysis and interaction effects

A mixed factorial ANOVA was conducted for SampEn and HFD to observe the main effects of time and group (control, IFN-β, or DMF). An interaction plot was created to visualize the effects.

#### 3.3.1 Interaction effects of time and treatment on sample entropy

**Figure 4:**
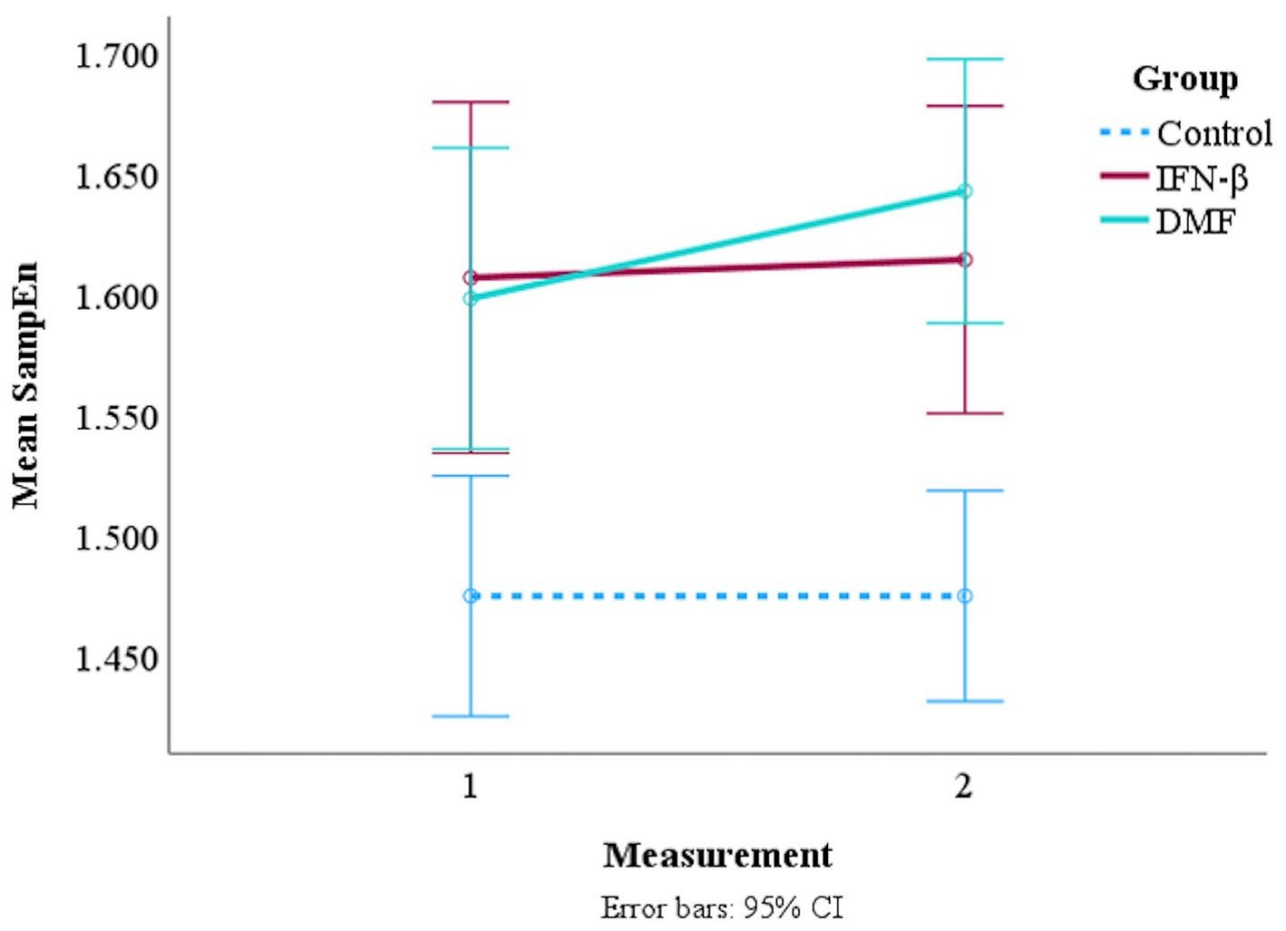
Interaction plot of mean SampEn over time across the treatment groups and the control group. *A second measurement for the control group was not collected. However, since no significant changes in resting-state EEG are expected in healthy subjects within 1 year, the control group is represented as constant in the interaction plot (Kondacs and Szabó, 1999).

Time did not have a significant effect, *F*(1, 172) = 1.905, *p* = 0.169, and an insignificant interaction effect of time and group was reported *F*(2, 172) = 1.336, *p* = 0.266. The results indicate that SampEn did not significantly change between the first- and second-time measurements across all groups, and the pattern of change over time was insignificant across all groups. Although the interaction plot (Figure 4) shows some level of interaction between IFN-β and DMF, the graph alone does not confirm any statistically significant interaction. Neither of the treatment groups intersected with the control group, indicating their trend is different from the control group. Accordingly, the results confirm no significance was reported when comparing the pattern of change in both treatment groups between measurements 1 and 2.

#### 3.3.2 Interaction effects of time and treatment on Higuchi’s fractal dimension

**Figure 5:**
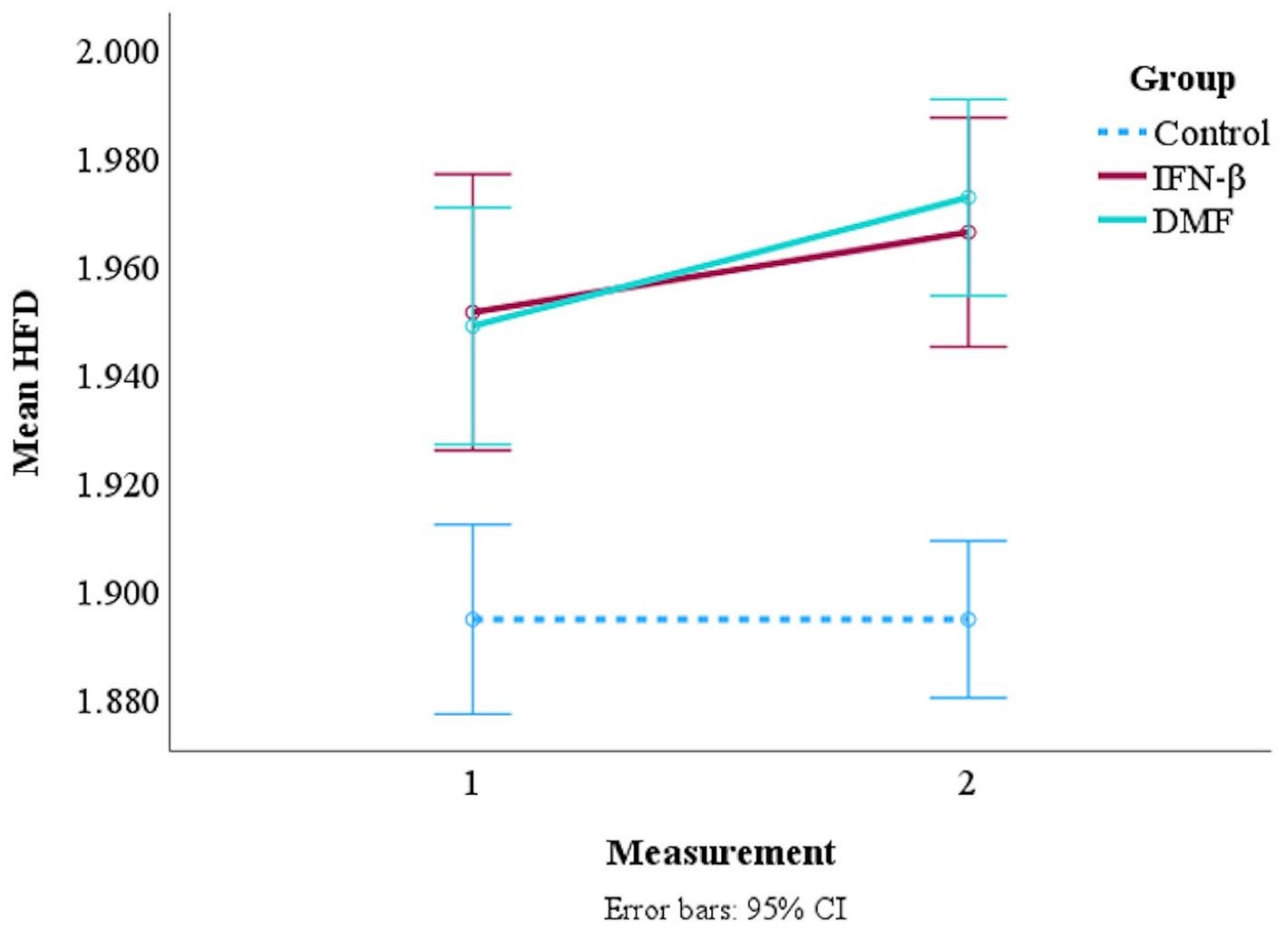
Interaction plot of mean HFD over time across the treatment groups and the control group. *A second measurement for the control group was not collected. However, since no significant changes in resting-state EEG are expected in healthy subjects within 1 year, the control group is represented as constant in the interaction plot (Kondacs and Szabó, 1999).

The mixed factorial ANOVA highlighted the main effects of time and group (control, IFN-β, or DMF). It yielded a significant effect for time *F*(1, 172) = 12.008, *p* < 0.001 and the interaction effect of time and group *F*(2, 172) = 4.384, *p* = 0.014. The results indicate that HFD significantly changed between the first- and second-time measurements across the treatment groups, and the pattern of change over time was significantly different. The detailed results are displayed in Table 4. The interaction plot (Figure 5) illustrates these findings. Both treatment groups saw an increase in their mean HFD in the second measurement, while the control group remained stable. The lines representing the two treatment groups did intersect, demonstrating some level of interaction. No interaction between either of the treatment group and the control group was observed. Hence, this also confirms the significance of the pattern of change in both treatment groups between measurements 1 and 2.

**Table 4:** Mixed ANOVA table results for HFD across groups and time measurements.

Source	Sum of squares	df	Mean square	*F*	*p*
Time	0.013	1	0.013	12.008	<0.001
Time*Group	0.010	2	0.005	4.384	0.014
Error(Time)	0.188	172	0.001	-	-

### 3.4 Diagnostic potential of complexity metrics

Due to the violation of homogeneity, Welch’s ANOVA was performed for the between-subjects effect at the first and second measurements for both SampEn and HFD. A Games-Howell *post hoc* test was conducted to identify significant differences between groups.

#### 3.4.1 Between-subjects effects of treatment on sample entropy

Welch’s ANOVA was conducted following the Levene’s test, which indicated a violation of homogeneity in the first measurement, *F*(2, 172) = 12.206, *p* < 0.001, and in the second measurement, *F*(2, 172) = 49.377, *p* < 0.001. The summary of the results is displayed in Table 5. Welch’s ANOVA revealed a significant effect of treatment in the first measurement, *F*(2, 97.945) = 6.446, *p* = 0.002, and the second measurement, *F*(2,104.188) = 13.059, *p* < 0.001. Games-Howell *post hoc* test (Table 6) revealed that IFN-β (M = 1.607, SD = 0.219) and DMF (*M* = 1.598, SD = 0.187) had significantly higher sample entropy values in the first measurement compared to the control group (*M* = 1.475, SD = 0.259). Specifically, the mean difference between IFN-β and the control group was −0.132, 95% CI [−0.240, −0.025], *p* = 0.012. DMF’s mean difference with the control group was −0.123, 95% CI [−0.214, −0.033], *p* = 0.005. There was no significant difference when comparing IFN-*β* and DMF in the first measurement (*p* = 0.978).

**Table 5:** Welch’s ANOVA for the effect of treatment group on sample entropy.

Measurement	Statistic	df1	df2	*p*
First measurement	6.446	2	97.945	0.002
Second measurement	13.059	2	104.188	<0.001

**Table 6:** Games-Howell *post hoc* comparisons for differences in sample entropy across treatment groups.

Dependent variable	(I) Group	(J) Group	Mean difference (I-J)	Std. Error	Sig.	95% Confidence Interval	
						Lower bound	Upper bound
SampEn first measurement	Control	IFN-β	−0.132	0.045	0.012	−0.240	−0.025
		DMF	−0.123	0.038	0.005	−0.214	−0.033
	IFN-β	Control	0.132	0.045	0.012	0.025	0.240
		DMF	0.009	0.043	0.978	−0.095	0.112
	DMF	Control	0.123	0.038	0.005	0.033	0.214
		IFN-β	−0.009	0.043	0.978	−0.112	0.095
SampEn second measurement	Control	IFN-β	−0.140	0.036	0.001	−0.225	−0.054
		DMF	−0.168	0.033	<0.001	−0.246	−0.090
	IFN-β	Control	0.140	0.036	0.001	0.054	0.225
		DMF	−0.028	0.028	0.563	−0.095	0.038
	DMF	Control	0.168	0.033	<0.001	0.090	0.246
		IFN-β	0.028	0.028	0.563	−0.038	0.095

For the second measurement, the Games-Howell *post hoc* test demonstrated that IFN-β (M = 1.614, SD = 0.138) and DMF (*M* = 1.643, SD = 0.121) had significantly higher sample entropy values in the second measurement compared to the control group (*M* = 1.475, SD = 0.259). In this measurement, the mean difference between IFN-β and the control group was −0.140, 95% CI [−0.225, −0.054], *p* = 0.001, and the mean difference between DMF and the control group was −0.168, 95% CI [−0.246, −0.090], *p* < 0.001. Like in the first measurement, there was no significant difference when comparing IFN-β and DMF in the first measurement (*p* = 0.563).

#### 3.4.2 Between-subjects effects of treatment on Higuchi’s fractal dimension

Like in SampEn, the Levene’s test confirmed a violation of homogeneity in the first measurement, *F*(2, 172) = 34.473, *p* < 0.001, and in the second measurement, *F*(2, 172) = 387.564, *p* < 0.001. Therefore, Welch’s ANOVA was conducted to determine the between-subjects effect in HFD values. A significant effect of treatment was observed in the first measurement, *F*(2, 103.306) = 9.799, *p* < 0.001, and in the second measurement, *F*(2,107.471) = 26.777, *p* < 0.001 was observed. A breakdown of the results is outlined in Table 7. The Games-Howell *post hoc* test (Table 8) was performed to identify where the significance lay. IFN-β (*M* = 1.951, SD = 0.065) and DMF (*M* = 1.949, SD = 0.064) had significantly larger HFD values in the first measurement compared to the control group (*M* = 1.895, SD =0 0.095). The mean difference between IFN-β and the control group was −0.057, 95% CI [−0.092, −0.022], *p* = 0.001. DMF’s mean difference with the control group was −0.054, 95% CI [−0.087, −0.022], *p* < 0.001. There was no significant difference when comparing IFN-β and DMF in the first measurement (*p* = 0.981).

**Table 7:** Welch’s ANOVA for the effect of treatment group on Higuchi’s fractal dimension.

Measurement	Statistic	df1	df2	*p*
First measurement	9.799	2	103.306	<0.001
Second measurement	26.777	2	107.471	<0.001

**Table 8:** Games-Howell *post hoc* comparisons for differences in Higuchi’s fractal dimension across treatment groups.

Dependent variable	(I) Group	(J) Group	Mean difference (I-J)	Std. Error	Sig.	95% Confidence Interval	
						Lower bound	Upper bound
HFD first measurement	Control	IFN-β	−0.057	0.015	0.001	−0.092	−0.022
		DMF	−0.054	0.014	<0.001	−0.087	−0.022
	IFN-β	Control	0.057	0.015	0.001	0.022	0.092
		DMF	0.003	0.014	0.981	−0.030	0.035
	DMF	Control	0.054	0.014	<0.001	0.022	0.087
		IFN-β	−0.003	0.014	0.981	−0.035	0.030
HFD second measurement	Control	IFN-β	−0.072	0.011	<0.001	−0.097	−0.046
		DMF	−0.078	0.011	<0.001	−0.103	−0.052
	IFN-β	Control	0.072	0.011	<0.001	0.046	0.097
		DMF	−0.006	0.004	0.170	−0.015	0.002
	DMF	Control	0.078	0.011	<0.001	0.052	0.103
		IFN-β	0.006	0.004	0.170	−0.002	0.015

Like the first measurement, the Games-Howell *post hoc* test demonstrated that IFN-β (*M* = 1.966, SD = 0.017) and DMF (*M* = 1.973, SD = 0.016) had significantly larger HFD values in the second measurement compared to the control group (*M* = 1.895, SD = 0.095). In this measurement, the mean difference between IFN-β and the control group was −0.072, 95% CI [−0.097, −0.046], *p* < 0.001, and the mean difference between DMF and the control group was −0.0780, 95% CI [−0.103, −0.052], *p* < 0.001. No significant difference was reported when comparing IFN-β and DMF in the first measurement (*p* = 0.170).

## 4 Discussion

Multiple sclerosis is a complex and progressive disease that is mostly diagnosed in young women. It impacts the central nervous system and causes various symptoms, such as deficits in complex attention, long-term memory, and processing speed (Chiaravalloti and DeLuca, 2008; Dobson and Giovannoni, 2019). It also reduces the brain’s ability to compensate for damage and cognitive reserve. It has been historically treated with immunosuppressant or immunomodulatory treatments, which must be ongoing to reduce inflammation (Dobson and Giovannoni, 2019). In line with Pritchard and Duke (1995), the high AC1 values highlight the deterministic nature of the EEG signals (Pritchard and Duke, 1995). Although a significant interaction between time and group was observed in the AC1 values, no other significant results were reported. This demonstrates that linear measures, such as AC1, capture only limited information regarding the complexity of EEG signals, emphasizing the need for nonlinear analyses. Thus, nonlinear analyses have been proven to be effective in the analysis of EEG data of MS patients (Hernandez et al., 2023). So, this study provides novel insights into pharmaceutical treatments’ effects on MS patients’ brain dynamics, as measured by sample entropy and Higuchi’s fractal dimension.

### 4.1 Evidence of complexity in MS EEG: sample entropy and Higuchi’s fractal dimension analysis

As mentioned, higher entropy values indicate that a system is complex, irregular, and unpredictable, often linked to a healthy system. On the other hand, lower entropy values indicate a more predictable and deterministic system (Duran et al., 2013; Pincus, 2006; Delgado-Bonal and Marshak, 2019). As for HFD, greater values indicate more complexity in the signal (Scarpa et al., 2017). Treatment was expected to have some level of impact on the complexity of the signal (Shalbaf et al., 2012; Thomasson et al., 2000).

In the study, the control, Interferon-β, and dimethyl fumarate groups displayed high SampEn and HFD values at each time measurement, supporting the hypothesis that an increase of complexity was observed. It is shown that both treatment groups displayed higher SampEn and HFD values when compared to the control group, suggesting that the MS patients were found to have a greater number of nonlinear segments. These findings were similar to those of Pezard et al. (2001), who reported higher entropy values compared to the control group when investigating Parkinson’s disease (Pezard et al., 2001). This further reveals MS patients treated with IFN-β and DMF have less predictable and more complex electrical activity compared to the controls (Pezard et al., 2001). The high nonlinearity can also be tied to the dimensionality of the electrical activity. Lachaux et al. (1997) described how dimensionality decreases if nonlinearity increases (Lachaux et al., 1997). This indicates that the MS patients treated with both treatments may have brain dynamics of a lower dimension (Pezard et al., 2001; Stam et al., 1994). Additionally, it has been noted that the increase in the complexity of EEG signals for MS patients is linked to the brain’s compensatory mechanisms and is indicative of the brain’s structural complexity (Wątorek et al., 2024). We can hypothesize that the higher complexity reported in the treatment groups could also be due to the brain’s adaptive response to the effects of the treatments, as they are responsible for the regulation of the immune system and reduction in inflammation (Jakimovski et al., 2018; Linker and Haghikia, 2016; Mills et al., 2018).

### 4.2 Distinct EEG patterns in MS treatments and sensitivity of complexity measures

There were no significant differences reported in the complexity characteristics of EEG signals between MS patients undergoing treatment with IFN-β and DMF at the first and second measurements, which rejects the hypothesis that patients treated with DMF will exhibit significant differences in complexity characteristics compared to patients treated with IFN-β. However, the second hypothesis was partially supported because the complexity characteristics (SampEn and HFD) of each treatment group differed significantly compared to the control group at each time measurement, as confirmed by Welch’s ANOVA and the Games-Howell *post hoc* test. These findings are backed by other studies that have concluded that nonlinear EEG measures can be sensitive to treatments (Pezard et al., 1998; Pezard et al., 2001; Wackermann et al., 1993).

In particular, as seen in Tables 6, 8, the mean differences in SampEn between each treatment group and the control group at the first and second measurements were higher than the mean differences observed in the same scenario for HFD. This indicates that SampEn demonstrated the highest sensitivity and the greatest predicted value in evaluating the effects of each treatment group compared to the control group, supporting our third hypothesis. These results suggest that treatments, such as IFN-β and DMF, impact the overall brain dynamics, as reflected by the higher sample entropy and Higuchi fractal dimension values.

### 4.3 Complexity EEG metrics as biomarkers for MS treatment effectiveness

Several studies (Hossain et al., 2022; Di Ieva et al., 2015) have investigated using nonlinear analysis in recognizing biomarkers in individuals with MS and healthy controls (Hernandez et al., 2023). Both entropy and fractal dimension have been used to either distinguish between conditions or differentiate between healthy and pathological brains in previous research (Marino et al., 2019; Smits et al., 2016; Zappasodi et al., 2014; Zappasodi et al., 2015; Bauer et al., 2011; Pezard et al., 2001). In this study, we aimed to explore whether sample entropy and HFD are reliable indicators for the progression of MS. We initially hypothesized that MS patients treated with IFN-β and DMF treatments would reveal significant and consistent changes over time relative to the control group.

Referencing Figure 2, it was observed that the initial measurements of SampEn and HFD demonstrated more dispersion compared to the second set of measurements. This observation could indicate the progression of MS over time, leading to more consistency in the results. Nevertheless, we determined that the hypothesis could only be partially supported because time and the interaction between time and treatment group significantly impacted only HFD and not SampEn. However, a significant increase from the first measurement to the second measurement was only observed in HFD values of the DMF group. Hence, an increase in signal complexity and positive neurophysiological changes can be attributed to DMF, which is reflected only in HFD. This finding is supported by Viglietta et al. (2015) and Vermersch et al. (2022). Viglietta et al. (2015) concluded that DMF reduces new and enlarging T2 lesions, gadolinium-enhancing lesions activity, and the number of new non-enhancing T2 lesions (Viglietta et al., 2015). Similarly, Vermersch et al. (2022) reported that more pediatric patients treated with DMF did not develop new or newly enlarging T2 lesions compared to those treated with IFN-β (Vermersch et al., 2022). These findings demonstrate the effectiveness of DMF in reducing disease activity and may explain the increase in EEG complexity over time compared to IFN-β. Although SampEn demonstrated the highest sensitivity and greatest predicted value, its responsiveness was limited when time was factored in. This finding signifies how HFD may be more responsive to temporal changes in EEG dynamics than SampEn.

### 4.4 Limitations and future research

There are a few limitations and opportunities for future research to note in this study. The first limitation is centered on the selection of the kmax parameter. Different methods of kmax parameter selection have been employed previously, but researchers have yet to agree on a universal method (Kesić and Spasić, 2016). Different parameter selection methods could alter the results. However, one of the most common methods was chosen in this study. This method was carried out by selecting the parameter where HFD reached a maximum or asymptote (Wanliss et al., 2021; Doyle et al., 2004; Wajnsztejn et al., 2016). Another possible limitation is the sample size of each treatment group. Increasing the sample size could have enhanced the results reported in this experiment. More specifically, the IFN-β treatment group had the lowest number of participants, and an increase in the number of MS patients on IFN-β could have highlighted clinically significant differences between the treatment groups.

There are several opportunities for future research. First, future studies could expand and balance the sample sizes for each treatment and collect longitudinal EEG data from the control group to strengthen the analysis and validate these findings. The next step in the study could be to analyze the EEG time series using multifractal methodology. This method helps quantify the data’s correlation structure through the set of scaling exponents, providing a deeper understanding of the data’s complexity (Wątorek et al., 2024). Furthermore, there are several methods to characterize complexity. One method is detrended fluctuation analysis (DFA), which is used to evaluate the Hurst exponent and can then be recalculated to determine the fractal dimension (Márton et al., 2014). Another method is the Lyapunov exponent, which is employed to identify chaotic behavior in the data and can be used to quantify data complexity (Yakovleva et al., 2020). The presented study investigates the effects of two immunomodulatory treatments; however, they aren’t the only treatments for multiple sclerosis. MS treatments include immunosuppressants (i.e., fingolimod), immunomodulatory therapies (i.e., IFN-β and DMF), and immune reconstitution therapies (i.e., alemtuzumab and cladribine) (Dobson and Giovannoni, 2019). Future studies could investigate the effects of immunosuppressants and immune reconstitution therapies on the brain’s dynamics via nonlinear analysis. These studies could use nonlinear analysis to investigate how these different treatment groups compare.

As reported by Hernandez et al. (2023), several articles have used machine learning algorithms in studying MS (Ahmadi and Pechenizkiy, 2016; Torabi et al., 2017; Kotan et al., 2019; Raeisi et al., 2020; Karaca et al., 2021; Karacan et al., 2022; Mohseni and Moghaddasi, 2022). Methods include feature extraction, feature selection, and feature classification, and these methods could allow researchers to swiftly search and analyze large datasets for potential biomarkers (Hernandez et al., 2023; Hossain et al., 2022). In future studies, researchers could build on this study’s approach by developing machine-learning methods that integrate MRI and functional magnetic resonance imaging (fMRI) to compare the efficacy of different MS treatments. This could further enhance the analysis by identifying trends and possible biomarkers more efficiently.

## 5 Conclusion

After demonstrating the limitations associated with lag-1 autocorrelation, we employed sample entropy and Higuchi’s fractal dimension to analyze the nonlinearity in electroencephalogram signatures of MS patients treated with Interferon-β and dimethyl fumarate. We have shown that patients undergoing each treatment exhibited more complex and less predictable brain activity when compared to the control group. SampEn demonstrated the highest sensitivity to treatment effects, whereas HFD revealed greater sensitivity when considering the effect of time.

Thus, these results have provided insights into how the effects of each treatment have a different impact on brain activity. They have furthered our understanding of the brain’s mechanics associated with MS. With the knowledge gathered here and on future investigations, current treatment strategies could be improved, and any benefits or limitations associated with these treatments could be disclosed. Thus, our study expands the scope of the analysis of EEG signatures of MS patients and paves the way for an alternative approach to analyzing treatment effectiveness.

## Data Availability

The raw data supporting the conclusions of this article will be made available by the authors, without undue reservation.

## Footnotes

^1^ https://www.mathworks.com/matlabcentral/fileexchange/50290-higuchi-and-katz-fractal-dimension-measures
